# fMRI Activity in the Macaque Cerebellum Evoked by Intracortical Microstimulation of the Primary Somatosensory Cortex: Evidence for Polysynaptic Propagation

**DOI:** 10.1371/journal.pone.0047515

**Published:** 2012-10-31

**Authors:** Teppei Matsui, Kenji W. Koyano, Keita Tamura, Takahiro Osada, Yusuke Adachi, Kentaro Miyamoto, Junichi Chikazoe, Tsukasa Kamigaki, Yasushi Miyashita

**Affiliations:** 1 Department of Physiology, The University of Tokyo School of Medicine, Tokyo, Japan; 2 Department of Physics, The University of Tokyo School of Science, Tokyo, Japan; University of Massachusetts Medical School, United States of America

## Abstract

Simultaneous electrical microstimulation (EM) and functional magnetic resonance imaging (fMRI) is a useful tool for probing connectivity across brain areas *in vivo*. However, it is not clear whether intracortical EM can evoke blood-oxygenation-level-dependent (BOLD) signal in areas connected polysynaptically to the stimulated site. To test for the presence of the BOLD activity evoked by polysynaptic propagation of the EM signal, we conducted simultaneous fMRI and EM in the primary somatosensory cortex (S1) of macaque monkeys. We in fact observed BOLD activations in the contralateral cerebellum which is connected to the stimulation site (i.e. S1) only through polysynaptic pathways. Furthermore, the magnitude of cerebellar activations was dependent on the current amplitude of the EM, confirming the EM is the cause of the cerebellar activations. These results suggest the importance of considering polysynaptic signal propagation, particularly via pathways including subcortical structures, for correctly interpreting ‘functional connectivity’ as assessed by simultaneous EM and fMRI.

## Introduction

Information processing in the brain is thought to be mediated through the interaction of cortical and subcortical regions in the brain [1]. Electrical microstimulation (EM) combined with functional magnetic resonance imaging (fMRI) is a powerful approach that allows *in vivo* mapping of such functionally interacting regions in the whole brain at a relatively high spatial resolution (∼1 mm). Several recent studies have demonstrated the feasibility of combining EM and fMRI (EM-fMRI) for *in vivo* mapping of cortico-cortical and cortico-subcortical connections in the macaque brain [2], [3], [4], [5], [6], [7]. To give physiological interpretation of a ‘functional connection’ detected by EM-fMRI, however, it is critical to understand the relationship between the ‘functional connection’ as detected by EM-fMRI and the underlying anatomical connections through which the EM effect can propagate. In particular, it is important to elucidate whether the effects of EM can spread polysynaptically to evoke blood-oxygenation-level-dependent (BOLD) responses in areas that are not directly connected to the site of stimulation.

In the case of thalamic EM, a recent EM-fMRI study showed that the effects of EM in the lateral geniculate nucleus could spread polysynaptically to the superior colliculus [6], [8]. For intracortical EM, previous studies using EM-fMRI with intracortical EM observed the activations produced by EM only in the cortical regions connected monosynaptically to the stimulated site [2], [9]. However, another study using EM-fMRI observed widely distributed, potentially polysynaptically mediated, activations produced by intracortical EM in the temporal cortex [5]. Therefore, it is not clear whether or not the effects of intracortical EM spread polysynaptically.

The cortico-cerebellar connection is a well-established polysynaptic connection (disynaptic for efferent and trisynaptic for afferent connections) [10] that is often used to test for the presence of polysynaptically mediated interactions (e.g. polysynaptic propagation of correlations in spontaneous BOLD signals [11] or transsynaptic neuronal circuit tracing using viral vectors [12]). In the present study we used the cortico-cerebellar connection to test for the possibility of polysynaptic propagation of the signal produced by intracortical EM. We found that the activity evoked by EM in the primary somatosensory cortex (S1) could in fact propagate polysynaptically to elicit BOLD responses in the contralateral cerebellum. Moreover, we showed that these responses were modulated as a function of the current amplitude used for the EM, supporting the notion that the intracortical EM is the cause of the cerebellar BOLD responses.

## Materials and Methods

Two male macaque monkeys were used (Monkey 1 and Monkey 2; macaca mulatta; 5.5 and 7 kg, respectively). Detailed procedures for EM-fMRI are described elsewhere [7] and will be described here only briefly. Prior to experimentation, monkeys were surgically implanted with custom-made MRI-compatible head-holding devices and MRI-compatible recording chambers (Crist Instruments, MD, USA) under aseptic condition. All procedures were performed in full compliance with the regulation of the University of Tokyo School of Medicine and the NIH guidelines for the care and use of laboratory animals. Part of the data is the same data used in our previous study [7] for a different purpose.

Monkeys were scanned with a 4.7-T MRI scanner (BioSpec 47/40, Bruker BioSpin, Ettlingen, Germany). For functional scans, a single-shot gradient-echo echo-planar imaging sequence was used (repetition time, 2.5 s or 3 s; echo time, 21 ms; flip angle, 80°; matrix size, 96×64; voxel size, 1.5×1.25×1.5 mm^3^; 25 or 33 axial slices, no gap). Anatomical images for each fMRI session were obtained using a T2-weighted fast spin-echo sequence (0.75×0.625×1.5 mm). High resolution anatomical images for individual monkey's anatomical templates were obtained, in different days, using a T1-weighted gradient-echo sequence (MDEFT; 0.5 mm isotropic).

At the beginning of each fMRI session, the monkeys were anesthetized with an intramuscular injection of medetomizine and midazolam (30 µg/kg and 0.3 mg/kg, respectively), and then a platinum-iridium microelectrode (0.2–0.3 MΩ; FHC, ME, USA) was inserted to the gray matter using a non-magnetic mini-manipulator system (Narishige, Tokyo, Japan) [13]. Multiunit neuronal activity elicited by tactile stimulation was used to guide and confirm the location of the microelectrode. After fixing the position of the microelectrode, the monkeys were transferred to the MRI scanner.

Throughout the fMRI scanning, anesthesia was maintained with continuous intravenous administration of propofol (6–8 mg/kg/hr) supplemented as needed by intramuscular injection of medetomidine [7], [14]. Heart rate and oxygen saturation were continuously monitored. Blood pressure was monitored between each of the functional runs. Body temperature was kept constant by using hot-water bags. Glucose-lactated Ringer's solution was given intravenously (5 ml/kg/hr) throughout the experiment.

Each fMRI run had a standard block-design consisting of 9 blank-blocks interleaved by 8 EM-blocks (30 s each). During each EM-block, 200 ms-electrical pulse trains were delivered at 1 Hz. Each pulse train was composed of biphasic current pulses (333 Hz) delivered in a monopolar configuration. One electric pulse consisted of 200 µs of negative phase followed by 200 µs of positive phase with a phase separation of 100 µs. A programmable constant current stimulator (SEN-7103, Nihon Kohden, Tokyo, Japan) was used for EM. A computer running the Presentation software (Neurobehavioral Systems, CA, USA) was used to synchronize fMRI scans with EM.

Image data were analyzed with SPM2 (http://www.fil.ion.ucl.ac.uk/spm) and in-house software written in MATLAB (Mathworks, Natik, MA). A high-resolution T1-weighted anatomical image was registered to bicomissural space to obtain a 3-dimensional template image for each monkey [15]. Functional images were realigned to the template image, with the interpolation to a 1 mm isotropic space, smoothed with a Gaussian kernel (1.5 mm full width at half maximum), and passed to voxel-wise statistical analyses based on general linear modeling (GLM). The significance level of activation was set at *P*<0.05 and corrected for multiple comparisons using the false discovery rate (FDR) [16]. Since the exact position of the stimulation electrode was variable across experiments, we analyzed data of individual experiments separately rather than averaging across experiments.

The magnitude of the cerebellar BOLD response was calculated for each fMRI run by averaging beta values obtained from the GLM analysis within the 4 mm diameter spherical region of interest (ROI). The center of the ROI was defined as the peak of BOLD activation in the contralateral cerebellum obtained with an EM of 500 µA. The spatial extent of the BOLD activation in the contralateral cerebellum was estimated, for each session, by counting the number of voxels in the contralateral cerebellum with *P*<0.001 (uncorrected). Statistical tests were performed with Statistics Toolbox in MATLAB (Mathworks, MA, USA).

### Ethics Statement

All procedures were performed in accordance with a protocol approved by the University of Tokyo Animal Care Committee (the permit number is 1923S001).

## Results

**Figure 1:**
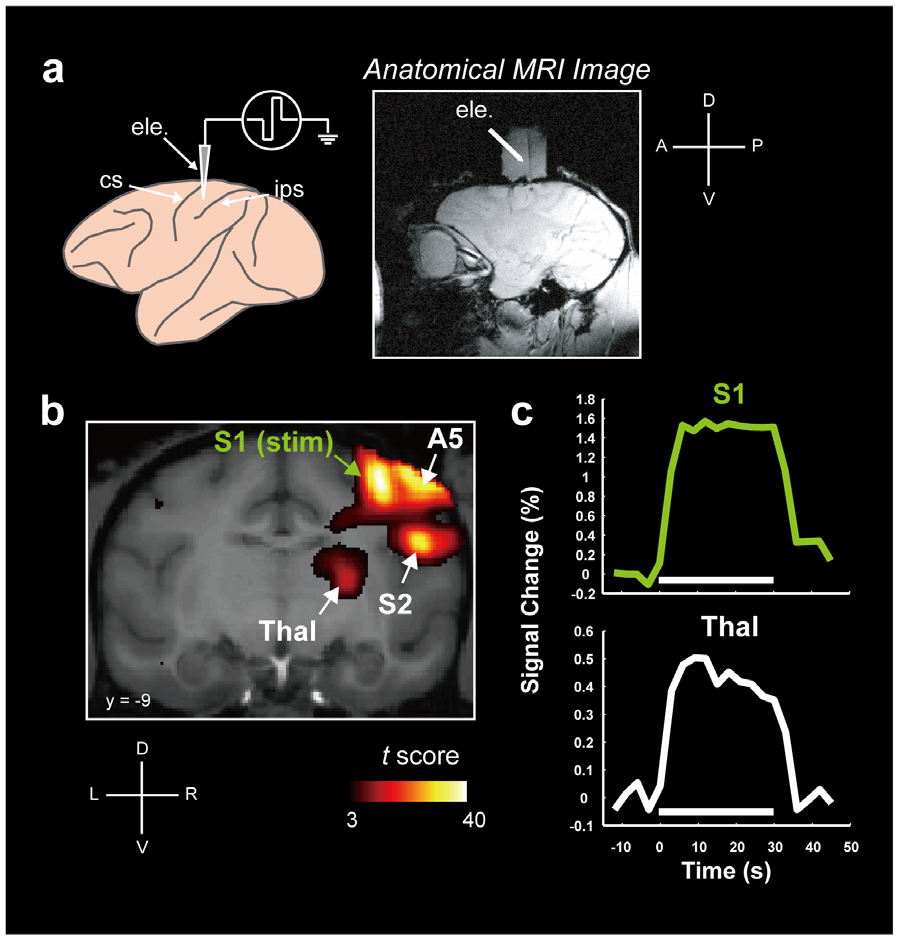
Simultaneous fMRI and electrical stimulation of S1. (**a**) Left panel, schematic drawing of a monkey brain with a microelectrode inserted in S1. Right panel, anatomical MRI image (FLASH) showing a monkey brain with a microelectrode inserted. ele, microelectrode. cs, central sulcus. ips, intraparietal sulcus. (**b**) Coronal sections of a representative *t*-score map of BOLD activation in Monkey 1 in one session (250 µA, 30 runs). In Monkey 1, right S1 was stimulated. A5, the area 5. Cb, cerebellum. S1, primary somatosensory cortex. S2, secondary somatosensory cortex. Thal, thalamus. stim, the site of EM. (**c**) Time courses of BOLD activations in S1 and Thal (30 runs, 240 EM blocks). Baseline signal [mean of 2 frames (6 sec) before the onset of EM block] was subtracted before averaging. White bars indicate 30 sec blocks of EM.

We conducted simultaneous intracortical EM and fMRI in two monkeys lightly anesthetized with propofol. A microelectrode for EM was inserted into the S1 gray matter posterior to the central sulcus (Fig. 1a). Monopolar electrical stimulation was given in 30 sec stimulation blocks interleaved by 30 sec blocks of no stimulation (see Methods for details). In both monkeys, EM evoked strong BOLD activation in S1 at and near the site of stimulation and in other cortical and subcortical areas known to have anatomical connections with S1, such as secondary somatosensory cortex and thalamus (Fig. 1b, Fig. S1a) [7]. The time courses of the BOLD signals in the activated regions clearly reflected the time course of EM (Fig. 1c, Fig. S1b).

**Figure 2:**
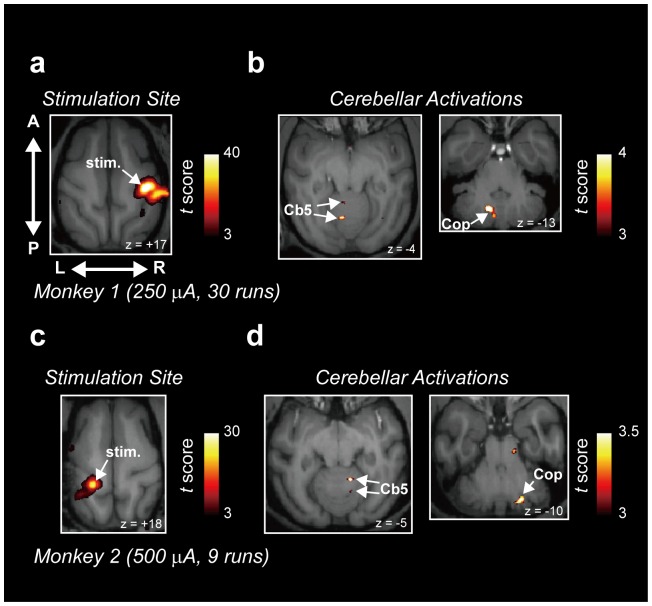
BOLD activations in the cerebellum induced by electrical stimulation of S1. (**a**)–(**b**) A representative *t*-score map of BOLD activation in Monkey 1 in one session (250 µA, 30 runs). (a) BOLD activation at the site of EM. In Monkey 1, right S1 was stimulated (arrow). (b) BOLD activations in the cerebellum. Cb5, cerebellar lobule V. Cop, copula myramidis. (**c**)–(**d**) A representative *t*-score map of BOLD activation in Monkey 2 in one session (500 µA, 9 runs). Conventions are the same as in (a) and (b). (c) BOLD activation at the site of EM. In Monkey 2, left S1 was stimulated (arrow). (d) BOLD activations in the cerebellum.

In addition to the activations found in the areas with monosynaptic connections to S1, multiple loci of activations were found in the contralateral cerebellum where only polysynaptic connections with S1 exist (Fig. 2). The peak cerebellar activations were found in lobule V and the copula pyramidis of the contralateral cerebellum (Fig. 2b), where somatosensory related activity has been reported [17], [18]. This pattern of cerebellar activation was reproducible in the other monkey, where left S1 was stimulated (Fig. 2c, d; Monkey 2, 9 runs, 500 µA; see Table 1 for the complete list of cerebellar activation peaks for the two monkeys). These results suggest that the effects of intracortical EM in S1 spread polysynaptically to produce multiple loci of BOLD activations in the contralateral cerebellum.

**Table 1:** List of BOLD activations in the cerebellum.

		Coordinates (mm)					
	Hemisphere	X	Y	Z	Volume (mm^3^)	*t* value	Area
**Monkey 1**	L	−5	−31	−13	65	5.29	Cop
	L	−9	−25	−7	15	4.37	Cb5/Cb6
	L	−3	−30	−4	27	4.22	Cb5
	L	−19	−29	−15	11	4.01	DPFl
	L	−2	−24	−4	8	3.44	Cb4
	L	−8	−30	−17	7	3.37	Cop
**Monkey 2**	L	−15	−38	−15	11	4.33	PM/Crus2
	L	−5	−39	−6	9	4.09	Cop
	R	9	−35	−10	14	3.9	Cop
	R	6	−25	−5	7	3.81	Cb5
	R	12	−33	−12	6	3.45	PM

List of the coordinates of the peaks of EM-evoked BOLD activations in the cerebellum for two monkeys (Monkey 1, 250 µA, 30 runs; Monkey 2, 500 µA, 9 runs). Significance level was set at *P*<0.05 (corrected). Activated regions with volumes ≥6 mm^3^ (2.1 original voxels) are included. Cb4, cerebellar lobule 4. Cb5, cerebellar lobule 5. Cb6, cerebellar lobule 6. Cop, copula pyramidis. Crus2, crus2 of ansiform lobule. DPFl, dorsal paraflocculus. PM, paramedian lobule. Anatomical areas are labeled by referring to the Paxinos et al. brain atlas [22].

**Figure 3:**
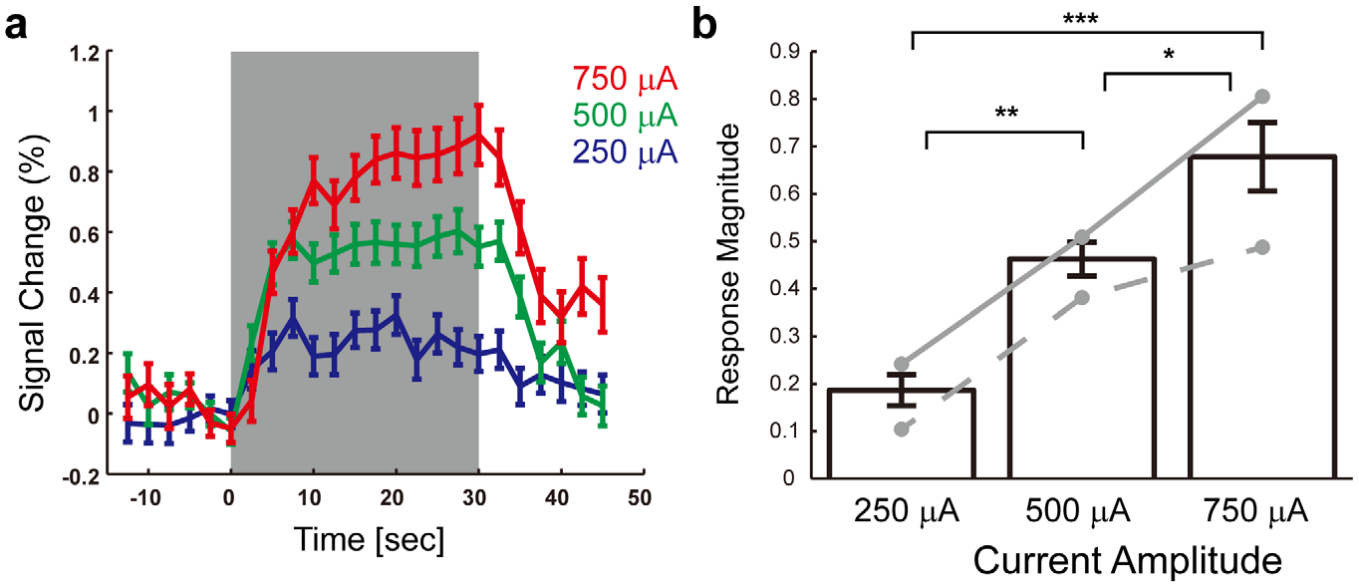
Current amplitude dependence of the response magnitude of the cerebellar activation. (**a**) Average time course of the cerebellar activation in response to an individual EM block. The shaded region indicates the EM block. For each time course, the baseline signal [mean of 2 frames (5 sec) before the onset of EM block] was subtracted before averaging. Error bars indicate standard error (SE). Red, 750 µA (240 blocks). Green, 500 µA (264 blocks). Blue, 250 µA (240 blocks). (**b**) Response magnitude for each current amplitude. Colored lines indicate data for individual monkeys (gray solid line, Monkey 1; gray dotted line, Monkey 2). Error bars indicate SE. *, *P*<0.02. **, *P*<0.0002. ***, *P*<10^−8^.

To examine the causal influence of EM on the cerebellar activations, we conducted five sets of experimental sessions in which the current amplitude for EM was varied (250 µA, 500 µA and 750 µA; 6–9 runs/condition in each set; 3 and 2 sets in Monkey 1 and Monkey 2, respectively). We first examined whether there was a relationship between the current amplitude used for EM and the response magnitude of the cerebellar activation. Time courses of BOLD response to individual EM blocks clearly showed that the magnitude of the response increased as the current amplitude increased (Fig. 3a) [Note that for each time course of an EM block, the baseline signal (mean of 5 sec before the onset of EM block) was subtracted before averaging across EM blocks]. Two-way analysis of variance (ANOVA) (Current Amplitude×Monkey) applied to the response magnitude of the cerebellar activation revealed a statistically significant main effect of Current Amplitude (*F_2,92_* = 24.18, *P*<10^−9^). The main effect of Monkey was also significant (*F_1,92_* = 12.36, *P*<0.0007), but there was not a significant interaction between the two factors (*F_2,92_* = 1.25, *P*>0.29). Comparing the data from the individual monkeys showed a similar trend (Fig. 3b, colored lines) suggesting that the difference in the results between the two monkeys was in the overall magnitude of the BOLD responses rather than any qualitative difference in the effects of the current amplitude. Post-hoc analysis revealed that the response magnitude at 250 µA EM was significantly smaller than that at 500 µA EM (*P*<0.004, post-hoc Tukey's test), and the response magnitude at 500 µA EM was smaller than that at 750 µA EM (*P*<0.02, post-hoc Tukey's test) (Fig. 3b). Thus, greater current amplitude applied to S1 produced larger BOLD responses in the contralateral cerebellum.

**Figure 4:**
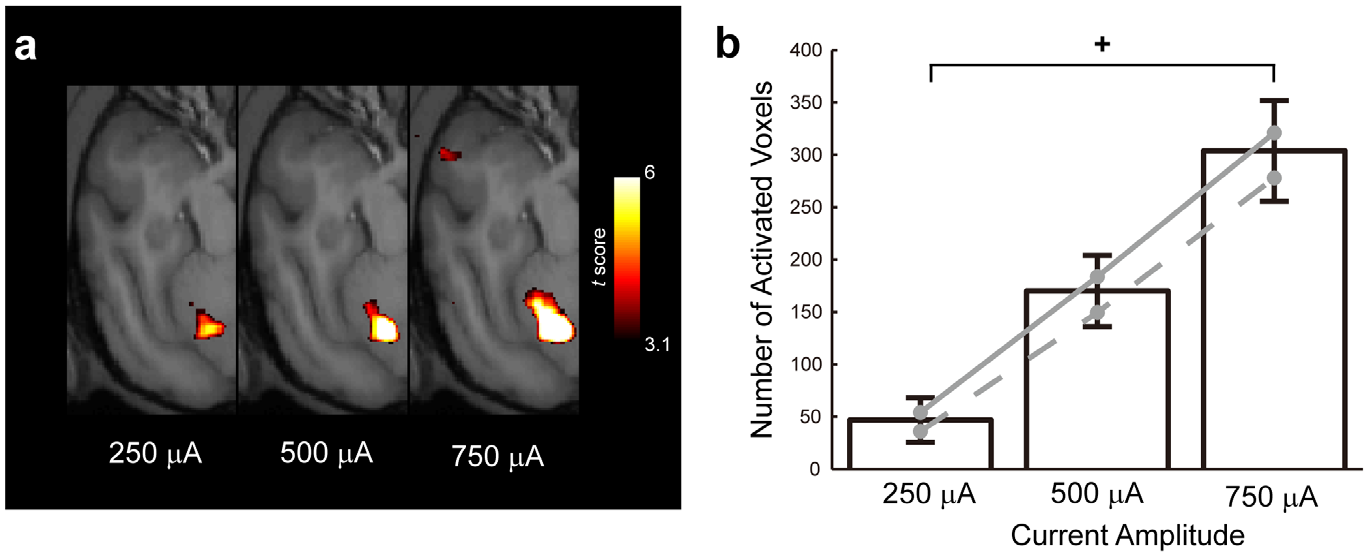
Current amplitude dependence of the size of the cerebellar activation. (**a**) Representative *t*-score maps (*P*<0.001, uncorrected) showing enlargement of activation with increasing current amplitude. Monkey 1, data from one session (250 µA, 6 runs. 500 µA, 9 runs. 750 µA, 6 runs). (**b**) Number of significantly activated voxels (*P*<0.001, uncorrected) in the contralateral cerebellum. Colored lines indicate data for individual monkeys (gray solid line, Monkey 1; gray dotted line, Monkey 2). Error bars, SE. +, *P*<0.005.

Furthermore, we examined whether there was a relationship between the spatial extent of the cerebellar activation and the current amplitude used for EM. Figure 4a shows representative cerebellar activations that increased in size as the current amplitude used for EM was increased. Total volume of significantly activated cerebellar voxels (*P*<0.001, uncorrected) was 46.8±47.3 mm^3^ (mean ± standard deviation), 170±76.0 mm^3^ and 304±107 mm^3^ for 250 µA, 500 µA and 750 µA respectively. Two-way ANOVA (Current Amplitude×Monkey) applied to the number of activated voxels in the contralateral cerebellum revealed a statistically significant main effect of Current Amplitude (*F_2,14_* = 9.44, *P*<0.007). The main effect of Monkey and the interaction between the two factors were not significant (*F_1,14_* = 0.44, *P*>0.5 for Monkey; *F_2,14_* = 0.02, *P*>0.9 for the interaction). Post-hoc analysis revealed that the number of activated voxels in the contralateral cerebellum was significantly smaller at 250 µA EM than 750 µA EM (*P*<0.005, post-hoc Tukey's test) (Fig. 4b). The same two-way ANOVA revealed significant main effect of Current Amplitude (*F_2,14_* = 11.2, *P*<0.004) but not of Monkey (*F_1,14_* = 1.85, *P*>0.2) and the interaction between the two factors (*F_2,14_* = 0.11, *P*>0.89). Post-hoc analysis revealed significantly smaller activation volume at 250 µA EM than at 750 µA EM (*P*<0.005, post-hoc Tukey's test). Thus, greater current amplitude applied to S1 resulted in activation of a larger volume of tissue in the contralateral cerebellum. Taken together, the current amplitude dependence of response magnitude and activated tissue volume support a causal influence of S1 EM in producing cerebellar BOLD activations.

## Discussion

Using intracortical EM and simultaneous fMRI in anesthetized macaque monkeys, we found BOLD responses in the cerebellum evoked by EM in S1. Since S1 and the cerebellum are only polysynaptically connected, the observed cerebellar activation suggests polysynaptic propagation of the activity produced by EM. To our knowledge, this is the first study showing evidence of polysynaptic propagation of the BOLD activity produced by intracortical EM in a current amplitude dependent manner. The fact that the effects of intracortical EM spread polysynaptically gives an important clue for properly interpreting previous (and future) results obtained by EM-fMRI. In particular, our results suggest it is important to take into account polysynaptic spread when inferring network structure of multiple cortical areas using EM-fMRI (e.g. [5]).

In our previous EM-fMRI study [7], the complete map of cerebellar activation could not be studied because cerebellum was covered only partially in the image volume. Importantly, it was unclear in the previous study whether cerebellar activations were localized in regions related to somatosensory system where activations evoked by EM in S1 should be expected. To address this problem, in the present study, we modified imaging parameters so as to cover the entire cerebellum within the image volume. Furthermore, to confirm the effect of EM on the cerebellar activation, we investigated current amplitude dependence of the cerebellar activations.

Cerebellar activations evoked by EM in S1 were indeed located in areas where somatosensory related activity has previously been reported. A previous human fMRI study reported BOLD activity in cerebellar lobule V evoked by tactile stimulation [18]. The copula pyramidis has also been reported to express c-fos and uptake of 2-deoxyglucose following stimulation of motor/sensory cortex in rat [17]. The shortest anatomical connection between S1 and the cerebellum is a disynaptic pathway in which S1 connects to the cerebellum through the pons [10]. In one animal (Monkey 1), a small but statistically significant activation in the ipsilateral pons was found (data not shown). This may indicate contribution of the orthodromic (feedforward) pathway, but activation in the pons in the other monkey was not clear (data not shown). However, it is possible that BOLD activation in the pons might be difficult to detect because of the presence of large physiological noise (e.g. cardiac pulsation) in the brain stem. Future studies using EM-fMRI combined with cardiac gating [19] may clarify the exact pathway producing the cerebellar activations.

Although the focus of the present study is on the presence of cerebellar activations, it should be mentioned that the distribution of EM-evoked activations in the whole brain was rather limited (e.g. lack of activation in the contralateral S1) despite the fact that many brain regions should be connected to S1 when polysynaptic connections were taken into account (for details of cortical and subcortical areas activated by S1 EM, see [7]). The extent of EM-evoked activations could have been limited partially by the use of anesthesia. Since we conducted electrode insertion and fMRI in the same experimental session, and because it was preferable to test all the current amplitude in each experiment, we chose to use anesthesia in order to engage monkeys in experiments lasting for a long time (typically more than 10 hours) without giving them excessive stress. Nevertheless, several lines of evidence suggest it is unlikely that the use of anesthesia significantly affected the present results. First, a previous study which conducted EM-fMRI in both anesthetized and awake monkeys reported that there was no difference in the pattern of EM-signal propagation [6]. Second, another study which conducted EM-fMRI in propofol anesthetized monkeys has reported widely distributed activation induced by EM of the superior colliculus [4]. Results from these studies suggest that the limited spatial extent of EM-evoked activations is more likely to be due to the nature of EM signal propagation (see below) rather than the use of anesthesia.

It has been proposed that the signal produced by EM may preferentially propagate via cortico-subcortical pathways [6]. Our results agree with and add to this hypothesis by showing that the signals evoked by intracortical EM can propagate polysynaptically via a cortico-subcortical pathway. A recent study reported widespread cortical and subcortical BOLD responses induced by EM in the deep cerebellar nuclei further supporting the notion that cortico-subcortical pathway is capable of propagating EM-evoked signal transsynaptically with high efficiency [20]. This is in contrast with cortico-cortical connections where transsynaptic propagation occurs exclusively in cortical regions monosynaptically connected to the site of intracortical EM [2], [9]. These previous studies coupled with our results suggest that care should be taken when interpreting ‘functional connectivity’ as assessed by EM-fMRI. Specifically, the role of subcortical areas that connect distinct cortical areas via cortico-subcortico-cortical pathways [21] need to be carefully considered when inferring the anatomical pathways that may mediate the effects of EM.

## Conclusions

Intracortical EM in S1 produced BOLD activity in the contralateral cerebellum. The magnitude and size of the cerebellar activation were dependent on the current amplitude used for EM. These results suggest that the signals produced by intracortical EM can spread polysynaptically at sufficient strength to produce BOLD responses in remote sites. Therefore, it is important to take into account polysynaptic pathways, especially those involving subcortical structures, when interpreting ‘functional connections’ revealed by EM-fMRI.

## Supporting Information

Figure S1 **EM-evoked BOLD activations in S1 and Thalamus of Monkey 2.** (**a**) Axial sections of a representative t-score map of BOLD activation in Monkey 2 in one session (500 µA, 9 runs). In Monkey 2, left S1 was stimulated. (**b**) Time courses of BOLD activations in S1 and Thal for Monkey 2 (9 runs, 72 EM blocks). Time courses were extracted from 2 mm-diameter spherical ROI centered at the peaks of activations. Baseline signal [mean of 2 frames (6 sec) before the onset of EM block] was subtracted before averaging. White bars indicate 30 sec blocks of EM.
(TIF)
