# Supplementary figures and images for: fMRI Activity in the Macaque Cerebellum Evoked by Intracortical Microstimulation of the Primary Somatosensory Cortex: Evidence for Polysynaptic Propagation
Source: PLoS One. 2012 Oct 31;7(10):e47515. doi: 10.1371/journal.pone.0047515 (PMC3485272; doi:10.1371/journal.pone.0047515)

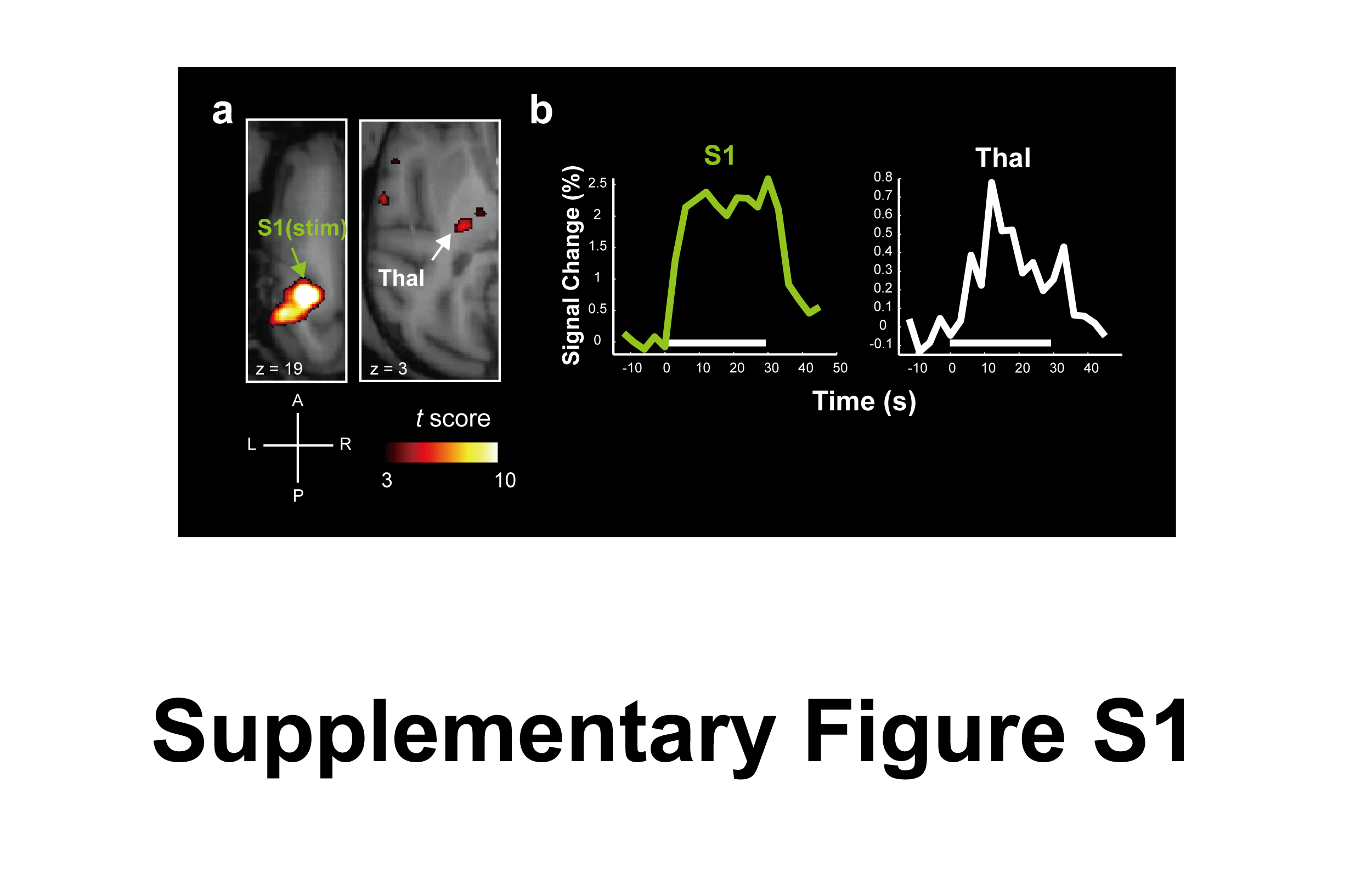

Supplement: Figure S1 — EM-evoked BOLD activations in S1 and Thalamus of Monkey 2. (a) Axial sections of a representative t-score map of BOLD activation in Monkey 2 in one session (500 µA, 9 runs). In Monkey 2, left S1 was stimulated. (b) Time courses of BOLD activations in S1 and Thal for Monkey 2 (9 runs, 72 EM blocks). Time courses were extracted from 2 mm-diameter spherical ROI centered at the peaks of activations. Baseline signal [mean of 2 frames (6 sec) before the onset of EM block] was subtracted before averaging. White bars indicate 30 sec blocks of EM. (TIF) [file pone.0047515.s001.tif]
